# Secondary untethering and re-untethering are both beneficial in adult tethered cord syndrome: a single-center cohort study

**DOI:** 10.1016/j.bas.2026.107441

**Published:** 2026-09-09

**Authors:** Luisa Mona Kraus, Maria Wostrack, Bernhard Meyer, Nicole Lange

**Affiliations:** a Department of Neurosurgery, School of Medicine and Health, Klinikum Rechts Der Isar, University Hospital of TUM, Ismaningerstraße 22, Munich, 81675, Germany; b Department of Neurosurgery, BG Trauma Center Murnau, Murnau, Germany

**Keywords:** Tethered cord syndrome, Retethering, Adhesiolysis

## Abstract

**Introduction:**

Adult tethered cord syndrome (TCS) represents a heterogeneous group of congenital and acquired conditions. While primary untethering is an established treatment, re-untethering remains controversial because of concerns regarding limited neurological benefit and increased complication rates.

**Research question:**

This study evaluates neurological outcomes after adults re-untethering surgery and identifies perioperative factors associated with clinical improvement.

**Methods:**

We performed a retrospective single-center cohort analysis of all patients surgically treated for TCS between 2008 and 2024. Patients were dichotomized into primary untethering and secondary re-untethering groups. Neurological status was assessed using the McCormick grade. Neurological improvement was defined as improvement of ≥1 McCormick grade. Postoperative complications and revision surgeries were recorded. Age and affected spinal level were analyzed as potential modifiers of outcome.

**Results:**

Seventy-one patients were included (median age 51.5 years); 42 underwent primary untethering and 29 re-untethering. Patients undergoing re-untethering presented with significantly worse baseline neurological status. Neurological improvement at discharge was more frequent after primary untethering (52.4% vs. 31.0%), but this difference reversed at follow-up, with higher improvement rates after secondary re-untethering (68.4% vs. 61.5%). Neither age nor affected spinal level significantly influenced neurological outcome or revision rates. Re-untethering was associated with more complex surgeries, and higher wound-healing complication rates.

**Discussion and conclusion:**

Despite worse baseline neurological status, re-untethering is not associated with inferior neurological outcomes compared with primary untethering. Neurological improvement appears to be a time-dependent process, and baseline impairment alone should not preclude surgical intervention. Re-untethering in adult patients should therefore be considered on an individual basis.

## Highlights

• Re-untethering can achieve meaningful neurological improvement despite higher baseline morbidity.
• Greater surgical complexity in re-untethering does not preclude favorable neurological outcomes.
• Earlier re-untethering may be associated with better neurological recovery.

## 1 Introduction

Tethered cord syndrome (TCS) is a complex condition characterized by an abnormal fixation of the spinal cord to surrounding meningeal structures, resulting in progressive neurological impairment. Typical clinical manifestations include pain, motor weakness, and neurogenic bladder dysfunction. Establishing the diagnosis can be challenging, as symptomatic patients do not always exhibit classical radiological findings (Aoun et al., 2019). Surgical untethering is considered a feasible and beneficial treatment in selected pediatric and adult patients (Hüttmann et al., 2001). Robust evidence supports primary untethering in patients with spinal dysraphism to halt or slow neurological deterioration (Koyanagi et al., 1997). Surgical treatment related risks may be spinal cord injury and the development of re-tethering. Favorable neurological outcomes are reported more frequently in pediatric patients, with improvement rates of approximately 70% compared with about 45% in adults (Shukla et al., 2025). Re-tethering rates of up to 30% have been described in pediatric patients with myelomeningocele (Hertzler et al., 2010). Given the young age at initial surgery, strategies of managing become increasingly relevant over time. Early re-untethering in this patient population has therefore been advocated as a viable therapeutic option (Ohe et al., 2000). Alternative surgical strategies, such as spinal column shortening, have been proposed to mitigate the risks associated with repeat untethering (McVeigh et al., 2021). Nevertheless, published outcomes of re-untethering remain largely unfavorable, and some authors even suggested conservative management in recurrent tethering (Sun et al., 2018). As there is little knowledge of the natural history of TCS in adults, treatment strategies are frequently extrapolated from pediatric cohorts. In addition to congenital etiologies, iatrogenic tethering may occur following intradural tumor resection across all age groups. Layered closure of the spinal meninges using pial sutures is commonly believed to reduce risk of postoperative tethering (Goto et al., 2003; Klekamp, 2013). Clinical symptoms may resemble that of typical TCS, data on the natural history and surgical outcomes of this acquired form of tethering remain sparse. Consequently, evidence-based guidelines for the management of re-tethering – particularly in adult patients – are lacking.

To contribute to the ongoing discussion whether re-untethering is futile or even harmful, we present our single-center experience on re-untethering in patients with neurologically impairing TCS.

## 2 Material and methods

### 2.1 Cohort definition

We performed a single-center, retrospective data bank analysis of all patients (adults only) treated surgically for any kind of TCS. The heterogeneous cohort included patients with congenital dysraphism or secondary TCS following previous surgery for dysraphism, spinal tumors, degenerative spinal disease, or infection.

In complex cases (n = 11), untethering was performed under intraoperative neurophysiological monitoring, including motor-evoked potentials and external anal sphincter electromyography. Expansile duraplasty using an equine pericardial graft was performed when deemed necessary (n = 12). All patients received physiotherapy from the postoperative period until hospital discharge.

The local ethics committee granted permission for this study. Since a retrospective chart review was performed no additional patient consent was acquired.

Inclusion criteria were:

- Preoperative imaging (MRI or CT/MR myelography) with diagnosis of tethered cord syndrome
- Intraoperative Untethering
- Age >18 years

Exclusion criteria were:

- Insufficient data on preoperative and postoperative performance status

Dichotomization into primary untethering and secondary (re-)untethering was based on the first index operation at our institution. Secondary (re-)untethering included re-untethering following untethering surgery or untethering as a complication of any other prior spinal surgery.

### 2.2 Primary endpoints

Primary endpoints were neurological outcome and complication rate.

Neurological performance status was assessed applying the McCormick grade at admission, at discharge and at follow-up (FU) if available. Last available follow-up was conducted either by telephone or during an in-person visit. Postoperative neurological improvement was considered if McCormick grade improved by ≥ 1 either postoperatively or until last available follow-up. Neurological improvement at discharge was used as the outcome variable in the univariable effect-modification analysis.

Any postoperative complication until last available FU was considered. Complications noted were re-tethering, hemorrhage, postoperative CSF leak, and infection.

### 2.3 Statistical analysis

GraphPad Prism (version 10.4.2) and IBM SPSS (version 30.0.0) were used for statistical analyses and data interpretation. All data was assessed for Gaussian distribution. Medians or means were further used accordingly. Medians were compared using the Mann-Whitney-U test. Proportions were compared using the Fisher's exact test. Statistical significance was assumed in p < 0.05.

Odds ratios (OR) with 95% confidence intervals (CI) were reported to descriptively illustrate effect size and direction. No formal statistical testing was performed. Univariate logistic analysis was used to test the effect of modifiers on binary primary endpoints. Continuous predictors were analyzed separately to maintain an appropriate events-per-variable ratio and minimize overfitting.

Group differences in primary outcomes across diagnostic entities were assessed using an exact permutation test of the chi-square test (Monte Carlo).

## 3 Results

### 3.1 Patient cohort

From 2008 to 2024 we treated 71 patients with tethered cord syndrome. Median age was 51.5 years (IQR 37-62); 36 patients were female, and 35 were male.

Patients were dichotomized into primary untethering and secondary (re-)untethering of any entity. Primary untethering was performed in 42 patients, secondary or re-untethering in 29 patients. Patient characteristics of both cohorts are depicted in Table 1.

**Table 1:** **Clinical characteristics of surgical groups** Primary untethering n = 42, re-untethering n = 29. *Median time to last available FU was 12 (IQR 3.8-51) months in primary untethering and 18 (IQR 9-47.5) months in secondary (re-)untethering. Percentages were calculated from all available FU data (n = 26 in primary untethering, n = 19 in secondary re-untethering). ^#^Six patients in with primary untethering and four patients with secondary (re-)untethering underwent more than one revision surgery and/or had more than one complication. ^α^ In one additional patient, the initial re-untethering was considered insufficient, necessitating re-re-untethering 10 days after surgery.

	Primary untethering	Secondary (re-) untethering	OR (95% CI)/p-value
Median age	54.5 (IQR 40-64.3)	46 (IQR 32-56)	
Mainly affected levels			
cervical	2 (4.8%)	3 (10.3%)	2.31 (0.36-14.77)
thoracic	4 (9.5%)	8 (27.6%)	3.62 (0.97-13.46)
lumbar	26 (61.9%)	16 (55.2%)	0.76 (0.29-1.98)
sacral	10 (23.8%)	5 (17.2%)	0.67 (0.20-2.21)
Neurological status			
**Median McCormick at presentation**	2 (IQR 2-2)	2 (IQR 2-3)	**p = 0.027**
back pain	19 (45.2%)	7 (24.1%)	0.39 (0.14-1.10)
radiating pain	18 (42.9%)	7 (24.1%)	0.42 (0.15-1.21)
motor deficit	7 (16.7%)	13 (44.8%)	***4.06 (1.36-12.12)***
sensory deficit	10 (23.8%)	12 (41.4%)	2.26 (0.81-6.29)
ataxia	7 (16.7%)	14 (48.3%)	***4.67 (1.57-13.89)***
genital numbness	3 (7.1%)	3 (10.3%)	1.50 (0.28-8.01)
neurogenic bladder dysfunction	10 (23.8%)	15 (51.7%)	***3.43 (1.24-9.48)***
**Postoperative improvement rate**	22 (52.4%)	9 (31.0%)	0.41 (0.15-1.10)
**Postoperative deterioration rate**	1 (2.4%)	7 (20.7%)	***9.91 (1.12-87.64)***
Follow-up improvement rate *	16/26 (61.5%)	13/19 (68.4%)	1.35 (0.38-4.78)
Complication rate			
wound healing disorder	2 (4.8%)	7 (24.1%)	***6.36 (1.22-33.31)***
re-tethering*	6 (23.1%))	3^α^(15.8%)	0.63 (0.14-2.87)
csf leak	7 (16.7%)	5 (17.2%)	1.04 (0.30-3.67)
hematoma	3 (7.1%)	2 (6.9%)	0.96 (0.15-6.16)
≥1**revision surgery due to complication^#^**	12 (28.6%)	14 (48.4%)	233 (0.87-6.27)
**Time to last revision (months)**	2 (0.0-21.8)	1 (0-12)	p = 0.719

Even though median preoperative McCormick grades showed identical values, statistical analysis yielded a significant distribution towards higher grades in patients with secondary (re)-untethering (p = 0.027) (Fig. 1).

**Fig. 1:**
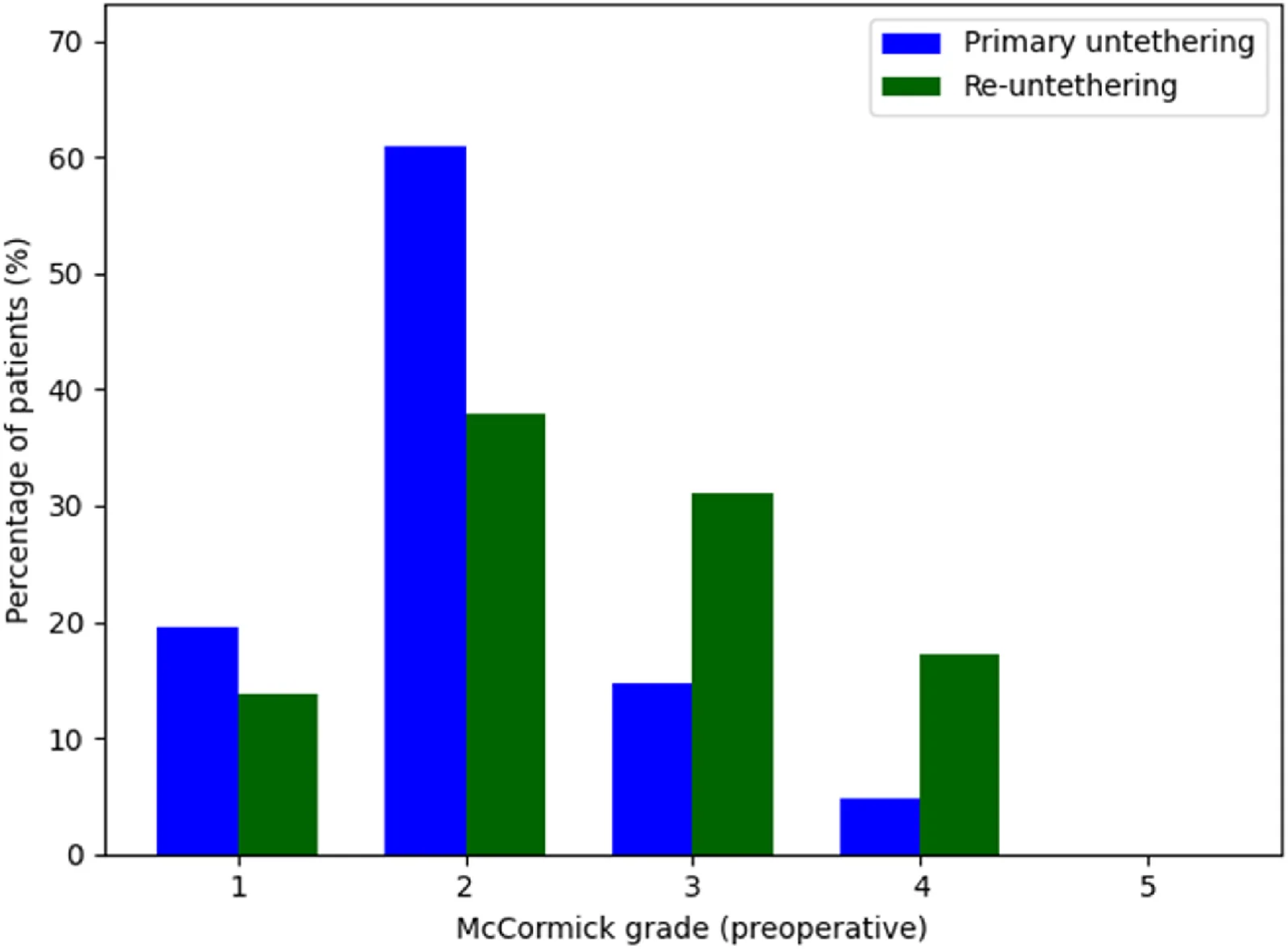
Preoperative neurological performance in both surgical groups.

Follow-up data were available for 45 of 71 patients, including 26 patients (57.8%) in the primary untethering group and 19 patients (65.5%) in the secondary (re-)untethering group. Of these 45 patients, 38 (84.4%) had a follow-up of at least 3 months, and 25 (55.6%) had a follow-up of at least 12 months. Among the latter, the median follow-up duration was 47.5 months (IQR 17.75–65.25).

Surgical strategies differed between the cohorts. Hemilaminectomy was the predominant approach in the primary untethering cohort (71.4%; Table 2), whereas secondary (re-)untethering was predominantly performed via laminectomy (55.2%). In addition, a greater number of spinal levels were treated in the secondary (re-) untethering cohort (median, 3 vs. 1 levels; p = 0.001), and operative time was significantly longer (180 vs. 131 min; p = 0.009).

**Table 2:** **Surgical approaches**^#^While adhesiolysis was defined as isolated untethering, in one patient in this cohort an addition gliding interface was used.

	Primary untethering	Secondary (re-) untethering	OR (95% CI)
Approach			
**Hemilaminectomy**	30 (71.4%)	11 (37.9%)	0.24 (0.09-0.67)
**Laminectomy**	7 (16.7%)	16 (55.2%)	6.15 (2.06-18.35)
**Laminoplasty**	5 (11.9%)	2 (6.9%)	0.55 (0.10-3.04)
**Multilevel**	20 (47.6%)	21 (72.4%)	2.89 (1.05-7.97)
**Median number of levels**	1 (IQR 1-2)	3 (IQR 1-3)	p = 0.001**
Kind of untethering			
**Adhesiolysis**	35^#^ (83.3%)	24 (82.8%)	0.96 (0.27-3.38)
**Expansive duraplasty**	7 (16.7%)	5 (17.2%)	1.04 (0.30-3.67)

**Surgery duration (minutes)**	131 (IQR 102-161)	180 (IQR 119-274)	p = 0.009*

### 3.2 Sub-cohort analysis of surgical groups

#### 3.2.1 Primary untethering

Diagnosis of patients who underwent primary untethering was spinal cord tethering in the presence of a thickened filum terminale in 21 patients (50.0%), split cord-syndrome in 6 patients (14.3%), intradural lipoma in 11 patients (26.2%) and spina bifida occulta in 4 patients (9.5%).

Postoperative neurological improvement did not differ across diagnostic entities (p = 0.870). Similarly, complication rates were comparable (p = 0.723).

#### 3.2.2 Secondary (re)-untethering

##### 3.2.2.1 Cohort description

Diagnosis of patients requiring re-untethering were prior surgery of any kind of intradural tumor in 13 patients (44.8%), re-tethering following primary untethering of any kind of dysraphism in 10 patients (34.5%), prior surgery for degenerative disease in one patient (3.4%), infection in three patients (10.3%) and trauma in two patients (6.94%). Median time from initial surgery to secondary (re-)untethering surgery was 71 months (IQR 21-317).

##### 3.2.2.2 Sub-cohort analysis of secondary tethering following intradural tumor surgery and re-tethering in dysraphism

Comparison of patients undergoing re-untethering following prior intradural tumor surgery and with those treated for congenital re-tethering demonstrated differences in age, pathology location and time between surgeries. Median age was higher in the tumor cohort (55 years (IQR 35-62) vs. 33 years (IQR 25.5-44.8)). Tumor-related tethering was frequently located outside the lumbar spine, whereas congenital pathologies predominantly involved the lumbar region (15.6% vs. 60.0%; OR 0.16, 95% CI 0.02-1.06). Time from initial surgery to index surgery at our institution was significantly shorter in the tumor sub-group (32 months (IQR 12-106) vs. 333 months (IQR 237-442), p = 0.0021)). Median preoperative McCormick grade was 2 in both groups. Motor deficits were observed in 46.2% vs. 30.0% of patients (OR 2.00, 95% CI 0.35-11.36), while neurogenic bladder dysfunction occurred in 38.5% vs. 40.0% (OR 0.86, 95% CI 0.16-4.47). Overall complication rates were comparable between groups (30.8% vs. 40.0%; OR 0.63, 95% CI 0.12-3.32).

Clinical outcomes were similar. Postoperative improvement was reported in 30.8% vs. 30.0% (OR 1.04, 95% CI 0.17-6.23), and improvement at last FU occurred in 60.0% vs. 85.7% (OR 4.00, 95% CI 0.25-63.95). Median FU duration was 12 months (IQR 7-16.5) in the tumor cohort and 47 months (IQR 10-62) in the congenital cohort.

### 3.3 Effect modification

Adhering to the stratification by surgical group the potential effect modifiers age and affected level were tested by univariate logistic analysis. As the median age over all patients was 51 years, this age was used as cut-off. Affected levels were dichotomized into cervical to thoracic and lumbosacral.

In both surgical groups the affected level did significantly influence neurological improvement or revision rate, whereas higher age in patients who underwent primary untethering was associated with higher odds of improvement (Table 3).

**Table 3:** **Effect modification of age and affected level on neurological improvement and revision rate** C/T = cervical/thoracic, LS = lumbosacral.

	Primary untethering		Secondary (re)-untethering	
	OR	95% CI	OR	95% CI
Neurological improvement				
age >51 vs. <51	*4.80*	*1.05-21.9*	1.89	0.39-9.24
C/T vs. LS	0.42	0.07-2.48	1.21	0.22-6.62
Revision rate				
age >51 vs. <51	1.16	0.28-4.75	1.13	0.26-4.94
C/T vs. LS	0.17	0.01-3.24	1.11	0.24-5.14

In patients who had secondary (re)-untethering higher latency to index surgery was associated with lower odds of neurological improvement (OR 0.93, 95% CI 0.87-1.00), while the number of operated levels did neither influence neurological outcome nor revision rate.

## 4 Discussion

Our study highlights the heterogeneity of conditions commonly summarized by neurosurgeons under the term of tethered cord syndrome. Rather than focusing exclusively on congenital TCS, this work also addresses the clinical history, neurological outcomes, and potential risks associated with surgical management of secondary tethering. Re-untethering is generally regarded as a complication following prior untethering surgery in patients with spinal dysraphism (Hertzler et al., 2010; Schoenmakers et al., 2003). Our data, however, suggest that a broader spectrum of retethering scenarios should be acknowledged. We therefore deliberately included patients who underwent untethering following degenerative spine or intradural tumor surgery. Presenting symptoms across these entities may vary depending on the affected level. Consequently, diagnosis of TCS in our cohort was established based on imaging findings in all patients.

As TCS predominantly affects pediatric patients, the management of re-tethering regarding long-term neurological outcome has long been a subject of debate (Klekamp, 2011). Ohe et al. advocated early re-untethering in formerly pediatric patients, reporting that early intervention was associated with improved neurological outcomes (Ohe et al., 2000). In addition to the time since index surgery, patient age has also been reported to influence neurological outcome, with younger patients demonstrating worse results (Al-Holou et al., 2009). Our findings differ somewhat from these observations. In our primary untethering cohort, we also report higher odds of neurological improvement in older patients. Simultaneously, the affected level did not appear to influence neurological outcome. Notably, patients undergoing re-untethering exhibited more severe neurological impairment at baseline, longer surgery duration, and anatomic complexity which may suggest limited surgical benefit. However, neurological improvement rate following primary untethering in our cohort was comparable to previously reported data (Hertzler et al., 2010). While re-untethered patients demonstrated a lower improvement rate and significantly higher deterioration rate at discharge, this was not statistically significant, and the trend was reversed at last available FU which was 12 months and 18 months, respectively. While this finding may be clinically relevant and should be communicated to appropriately manage patients’ expectations before surgery, it should be interpreted with caution given the limited follow-up data available. Whereas most published studies focus on adult FU of patients treated for TCS in childhood, data on the short- and long-term effects of primary and re-untethering procedures in the adults remain scarce. As the literature reports ranging improvement and re-tethering rates in adults after surgery for dysraphism (Klekamp, 2011), the need for long-term FU is undeniable. We acknowledge that follow-up duration was limited in a proportion of our patients; however, in our cohort, 55.6% of patients with available FU data had a FU duration of at least 12 months.In our primary untethering cohort, the predominant diagnoses were a thickened filum terminale and intradural lipomas that became symptomatic later in life. Consistent with the existing literature (Hertzler et al., 2010), these patients most commonly present with back pain and radiating pain. In contrast, patients with secondary tethering predominantly exhibited motor and sensory deficits as well as neurogenic bladder dysfunction.

This secondary tethering sub-cohort did not only comprise patients with re-tethering following untethering in dysraphism but was particularly heterogeneous. Interpretation of data on neurological improvement need therefore to be considered in the light of the underlying pathology. Patients developing TCS as a delayed complication following intradural spinal tumor resection present a distinct subgroup requiring separate consideration. In these cases, neurological deficits can be result of the actual tumor growth. Furthermore, postoperative scarring, and inflammation may lead to tissue overgrowth and subsequent tethering. The use of pia sutures following tumor resection has been proposed as a strategy to lower tethering rates (Klekamp, 2013). Compared with patients undergoing re-untethering after surgery of congenital TCS, the pathology in this subgroup was predominantly located in the thoracic spine, which may explain atypical neurological presentation such as ataxia rather than neurogenic bladder dysfunction. Despite differences in pathology and symptom distribution, neurological impairment at presentation was comparable, and both cohorts benefited from surgical intervention. The odds of neurological improvement decreased by 7% per year, thus promoting early intervention. Diagnosing TCS in patients with prior intradural tumor surgery remains challenging, as alternative causes of neurological deterioration – particularly tumor recurrence – must be carefully excluded. As expected, revision rates, especially early complications such as wound-healing disorders or hematoma were higher in this subgroup. In contrast, rates of re-tethering and CSF leakage were lower than those observed after primary untethering. Importantly, despite early postoperative complications, these patients demonstrated increasing neurological improvement during FU after discharge. This effect was independent of age, with even older – and naturally more physically disabled – patients benefiting from re-untethering surgery.

Overall, re-untethering was not associated with inferior neurological outcomes or disproportionately higher complication rates despite worse neurological baseline status and more complex intraoperative anatomy. Moreover, neurological improvement in more severely impaired patients with re-tethered cord syndrome seemed to be a time-dependent process. Thus, baseline neurological performance does not preclude surgical benefit; rather, clinical trajectory and symptom progression appear to be the most relevant determinants of operative potential.

### 4.1 Limitations

This study is limited by its monocentric and retrospective character as well as limited patient number. Power of our sub-cohort analyses was therefore limited. FU rate varied across sub-cohorts and long-term FU-rate was low. Neurological performance at a median last available FU at 12 and 18 months was only available in 65.5% and 55.8% thus limiting reliable long-term conclusions. Time to FU was also used as a proxy for time-to-improvement.

## 5 Conclusion

Tethered cord syndrome includes a variety of diagnoses in different age groups. While primary untethering is an established concept in pediatric as well as adult patients, data on re-untethering is contradictory. Patients that are suitable for re-untethering are not homogenous. We show that re-untethering in patients of younger and older age and various diagnosis can lead to neurological improvement and should therefore be considered on an individual basis, while accounting for higher revision rates and a potentially longer time to neurological recovery.

Declaration of generative AI and AI-assisted technologies in the manuscript preparation process.

During the preparation of this work the author(s) used ChatGPT5.2 (OpenAI, San Francisco, California, USA) to assist with spelling, grammar, and style corrections. After using this tool/service, the author(s) reviewed and edited the content as needed and take(s) full responsibility for the content of the published article.

## Ethics declaration

The authors have NOT obtained informed consent from participants or their legal representatives. As a retrospective chart review was performed no additional consent was required.

This study was performed in compliance with relevant laws, regulatory frameworks and guidelines where the research took place. This study was approved by the TUM School of Medicine and Health. (Approval No. 2026-174-S-CT)

## Declaration of competing interests

The authors declare the following financial interests/personal relationships which may be considered as potential competing interests: Given his role as Senior Editorial Board member, Professor Bernhard Meyer had no involvement in the peer review of this article and had no access to information regarding its peer review. Full responsibility for the editorial process for this article was delegated to another journal editor. If there are other authors, they declare that they have no known competing financial interests or personal relationships that could have appeared to influence the work reported in this paper.
